# Nutritional, functional, and microbial qualities of legume-based flour blends processed by SMEs in Zambia and Malawi compared to standard Corn-Soy Blend Plus (CSB +): a cross-sectional study

**DOI:** 10.1186/s40795-025-01034-0

**Published:** 2025-03-06

**Authors:** Patrick Ndovie, Smith G. Nkhata, Numeri Geresomo, Robert Fungo, Vincent Nyau, Richard Banda, Justice Munthali, Martha Chizule, Nellie Manda

**Affiliations:** 1 Department of Human Nutrition and Health, Lilongwe University of Agriculture and Natural Resources, Bunda Campus, P.O. Box 219, Lilongwe, Malawi; 2 Department of Agriculture and Food Systems, Natural Resources College, Lilongwe University of Agriculture and Natural Resources, P.O. Box 143, Lilongwe, Malawi; 3 School of Food Technology, Nutrition & Bioengineering, Makerere University, P.O. Box 7062, Kampala, Uganda; 4 Alliance of Bioversity International & CIAT, Agricultural Research Station, P.O. Box 158, Lilongwe, Malawi; 5 Department of Food Science & Nutrition, School of Agricultural Sciences, University of Zambia, P. O. Box 32379, Lusaka, Zambia

**Keywords:** Legumes, Flour blends, Proximate analysis, Functional properties, Microbial characteristics, RDA, Food safety, Malawi, Zambia

## Abstract

**Background:**

Legumes enhance food security in developing countries, necessitating an understanding of their properties. This study examined the nutritional, functional, and microbial qualities of legume-based flour blends from Small and Medium Enterprises (SMEs) in Malawi and Zambia. SMEs were chosen for their key role in local food production, distribution, and complementary food supply.

**Method:**

A total of 36 legume-based flour blend samples were collected using snowball sampling, consisting of 21 samples (7 sets of 3 similar samples) from SMEs in Zambia and 15 samples (5 sets of 3 similar samples) from SMEs in Malawi. Samples were analyzed for proximate composition, energy, iron, and zinc content. The nutritional contributions to the Recommended Dietary Allowances (RDA) for children aged 1–3 years were assessed. Additionally, functional properties such as water-holding and oil-holding capacities were measured. Microbial analysis was performed, and the data were statistically analyzed to determine significance (*p* ≤ 0.05).

**Results:**

Our findings revealed substantial variability in the nutritional content of these flour blends. Protein content ranged from 9.4% to 41.5%, carbohydrates from 8.1% to 71.3%, crude fat from 2.3% to 26.8%, and crude fiber from 6.2% to 35.2%. Iron and zinc levels also varied significantly, from 2.9 to 21.9 mg/100 g and 2.2 to 5.2 mg/100 g, respectively. These inconsistencies highlight a lack of standardization in nutrient content for blends intended for infant feeding. When prepared as 96 g porridge servings for children aged 1–3 years, the blends provided notable contributions to the Recommended Dietary Allowance (RDA). However, their nutrient levels were generally lower compared to the standard Corn-Soy Blend Plus (CSB +). The flour blends also showed variations in physico-functional properties, and some had microbial loads exceeding 250 cfu/g, reflecting inadequate hygiene practices during processing.

**Conclusion:**

To enhance their products, SMEs should ensure that their flour blends meet both nutritional and safety standards while striving to match or surpass the nutrient content of CSB + to remain competitive in the market.

## Introduction

Legumes provide essential nutrients for millions of people in developing countries [1]. They provide protein, calcium, iron, phosphorus, and other key minerals necessary for human health [2]. Beyond their nutritional value, legumes are versatile, serving as food for humans, feed for livestock, or raw materials in processed products [3]. In many developing countries, legumes have been strategically used to combat malnutrition, particularly protein-energy malnutrition (PEM), among vulnerable groups such as children, the elderly, and people living with HIV/AIDS [4]. Furthermore, legumes are recognized as potent nutraceuticals, offering health benefits such as the prevention of cancer, diabetes, digestive disorders, and cardiovascular diseases [5, 6].

Despite these benefits, the presence of anti-nutritional factors, such as phenolic compounds and oxalates, limits the bioavailability of key nutrients in legumes. Processing techniques are necessary to reduce these anti-nutrients and enhance nutrient availability [7]. For example, processing alters the polyphenolic profiles in legumes like common beans and Bambara groundnuts, improving their nutritional quality [8]. Additionally, legumes possess functional properties, such as starch and dietary fiber, that make them valuable ingredients for functional food development. These properties include a high content of resistant starch and dietary fiber, which contribute to a low glycemic index and hypoglycemic effects, making legumes suitable for managing diabetes and related health conditions [9, 10].

However, several factors limit the consumption and marketability of legumes, including their distinct beany flavor, short shelf life, and susceptibility to microbial contamination. Fungal spoilage is a significant challenge, particularly for legume-based products [11]. Addressing these challenges is essential to maximize the potential of legumes in improving nutrition and health outcomes.

Small and medium enterprises (SMEs) play a vital role in the economic development of countries, particularly in developing regions [12]. SMEs are crucial contributors to agricultural production, accounting for over 85% of Africa’s legume production from farms with less than 20 hectares [13, 14]. In countries like Ghana, Nigeria, and Kenya, SMEs produce more than 30% of legumes, such as beans, cowpeas, groundnuts, and soybeans [15]. These SMEs are integral to achieving Sustainable Development Goals (SDGs) like SDG 9 (Industry, Innovation, and Infrastructure), SDG 2 (Zero Hunger), and SDG 12 (Responsible Consumption and Production). These goals are essential to sustainable agricultural practices but often receive limited attention due to a focus on trade, agro-industry, and agriculture policies [16].

The diversity of food systems and diets worldwide is largely shaped by rural development and farming, which directly impacts nutrition and health outcomes [17]. SMEs can improve nutrition and health by making nutritious foods more accessible to consumers. However, SMEs face significant challenges such as limited access to credit, market volatility, price instability, and regulatory constraints [18, 19]. This research aims to assess the nutritional, functional, and microbial properties of legume-based flour blends produced by SMEs in Malawi and Zambia and evaluate their contribution to the recommended dietary allowance (RDA) for children aged 1–3 years old. The study contributes to food security and public health by providing evidence on the nutritional adequacy and safety of SME-produced complementary foods, informing policymakers, and guiding SMEs in improving product quality and formulation.

## Materials and methods

### Sources of legume-based flour blends

Legume-based flour blends for this study were obtained from small and medium enterprises (SMEs) operating in Malawi and Zambia. The samples were collected either directly from the processing facilities of SMEs or purchased from retail outlets, including supermarkets and grocery stores in urban areas.

Malawi and Zambia were chosen for this study as they are focal areas in a USAID-funded project aimed at enhancing food security and supporting SMEs in agricultural value chains. These countries are also prominent producers of legumes, making them suitable for assessing the quality and safety of legume-based products within the region.

### Sampling techniques, sample size, and transportation

A snowball sampling approach was used to identify SMEs producing legume-based flour blends. Initially, a target of 30 samples (15 from each country) was set, but the final collection included 36 samples—21 from Zambia (7 sets of 3 similar samples) and 15 from Malawi (5 sets of 3 similar samples). Both Zambian and Malawian processors were included due to their role as representatives of small-scale processing facilities crucial to the local economy. These were selected because they were legume-based flour blends processors. In Malawi, samples were collected from three major cities—Mzuzu, Lilongwe, and Blantyre—to capture geographic and market diversity.

Samples were packed in airtight, food-grade containers to prevent contamination during transportation. Refrigerated samples were kept in portable coolers containing ice. Upon arrival, samples were stored in the Food and Nutrition Laboratory at the University of Zambia (UNZA) and later transferred to the Food Analysis Laboratory at Lilongwe University of Agriculture and Natural Resources (LUANAR) for analysis.

### Proximate analysis

The proximate composition of the flour blends, including moisture, crude protein, crude fat, total ash, and crude fiber content, was determined using standard methods outlined by the Association of Official Analytical Chemists [22]. Carbohydrate content was calculated by difference, as shown in Eq. 1 [20]

1 $$\mathrm{Carbohydrate}\;\mathrm{content}\;\left(\%\right)=100-\left[\mathrm{moisture}\;(\%)\;+\;\mathrm{crude}\;\mathrm{protein}\;(\%)\;+\;\mathrm{crude}\;\mathrm{fat}\;(\%)\;+\;\mathrm{total}\;\mathrm{ash}\;(\%)\;+\;\mathrm{crude}\;\mathrm{fiber}\;(\%)\right].$$

### Determination of micronutrients (Iron and Zinc)

Iron and zinc concentrations were determined using atomic absorption spectrometry (AAS). Samples were digested with nitric acid, and their absorbance was measured at specific wavelengths: 248.3 nm for iron and 213.9 nm for zinc. Standard calibration curves for each micronutrient were prepared, following methods described by Lazarte et al. [21] and AOAC [22], to ensure accurate quantification.

### Nutritional contribution of flour blends

The contribution of the flour blends to the recommended dietary allowances (RDA) for children aged 1–3 years was assessed based on a porridge formulation. The preparation followed a standard flour-to-water ratio of 20% solids, using two daily servings of approximately 48–50 g of flour per serving, as recommended for Corn-Soy Blend Plus (CSB +) porridge [23]. The total daily flour intake was calculated using Eq. 2 and 3 [24]:

2 $$Total\;amount\;of\;flour\;(\%)\;=\frac{50\mathrm g\;\mathrm{of}\;\mathrm{CSB}\;\mathrm{flour}}{250\mathrm g\;\mathrm{of}\;\mathrm{water}}\times100$$

3 $$\mathrm{Total}\;\mathrm{flour}\;\mathrm{consumed}=\mathrm{Total\;amount\;of\;flour}\;(\%)\;\mathrm\times\mathrm{total\;servings}/\mathrm{day}=48-50\mathrm g\;\mathrm{flour}\;\times 2\;\mathrm{servings}\;=96-100\;\mathrm{grams\;in\;two\;servings}$$

Further, Eq. 4 was used to determine the total amount of nutrients to be provided by every flour blend used [25].

4 $$Total\;nutrients=\frac{\mathrm{Total}\;\mathrm{flour}\;\mathrm{consumed}\;\times\;\mathrm{nutrients}\;\mathrm{content}\;\mathrm{of}\;\mathrm{flour}}{100}$$

### Functional properties analysis

The functional properties of the flour blends, including water-holding and oil-holding capacities, were assessed to determine their suitability for developing functional food products. Water-holding capacity was determined by mixing 2 g of flour with 10 ml of distilled water, followed by centrifugation at 3500 rpm for 30 min, as described by Shevkani et al. [26]. Oil-holding capacity was similarly determined by mixing 2 g of flour with 10 ml of vegetable oil, vertexing for 5 min, and measuring the oil absorbed. These properties are critical as they influence the texture, stability, and overall quality of food products. Oil holding capacity (OHC) and Water holding capacity was calculated as described in Eq. 5 and 6 respectively.

5 $$WHC=\frac{\mathrm{Volume}\;\mathrm{of}\;\mathrm{water}}{\mathrm{Weight}\;\mathrm{of}\;\mathrm{the}\;\mathrm{sample}}\times100$$

6 $$OHC=\frac{\mathrm{Volume}\;\mathrm{of}\;\mathrm{oil}}{\mathrm{Weight}\;\mathrm{of}\;\mathrm{the}\;\mathrm{sample}}\times100$$

### Microbial analysis

The microbial quality of the flour blends was assessed through total viable colony counts. About 25 g of each sample was homogenized in 225 ml of peptone water, and serial dilutions (10⁻^1^ to 10⁻^5^) were prepared. A 1 ml aliquot from each dilution was plated on nutrient agar and incubated at 37 °C for 24 h and at 25 °C for five days, following the methods of Rajapaksha et al. [27]. Colonies were counted using a digital colony counter to determine microbial load.

### Statistical analysis

Data from proximate composition, functional properties, and microbial analyses were subjected to statistical analysis using analysis of variance (ANOVA) in SPSS version 26. Differences among means were tested for statistical significance (*p* ≤ 0.05) using Tukey’s post-hoc test. Comparisons were made across the different flour blends and against the CSB + standard.

## Results

### Proximate composition of flour blends

**Fig. 1:**
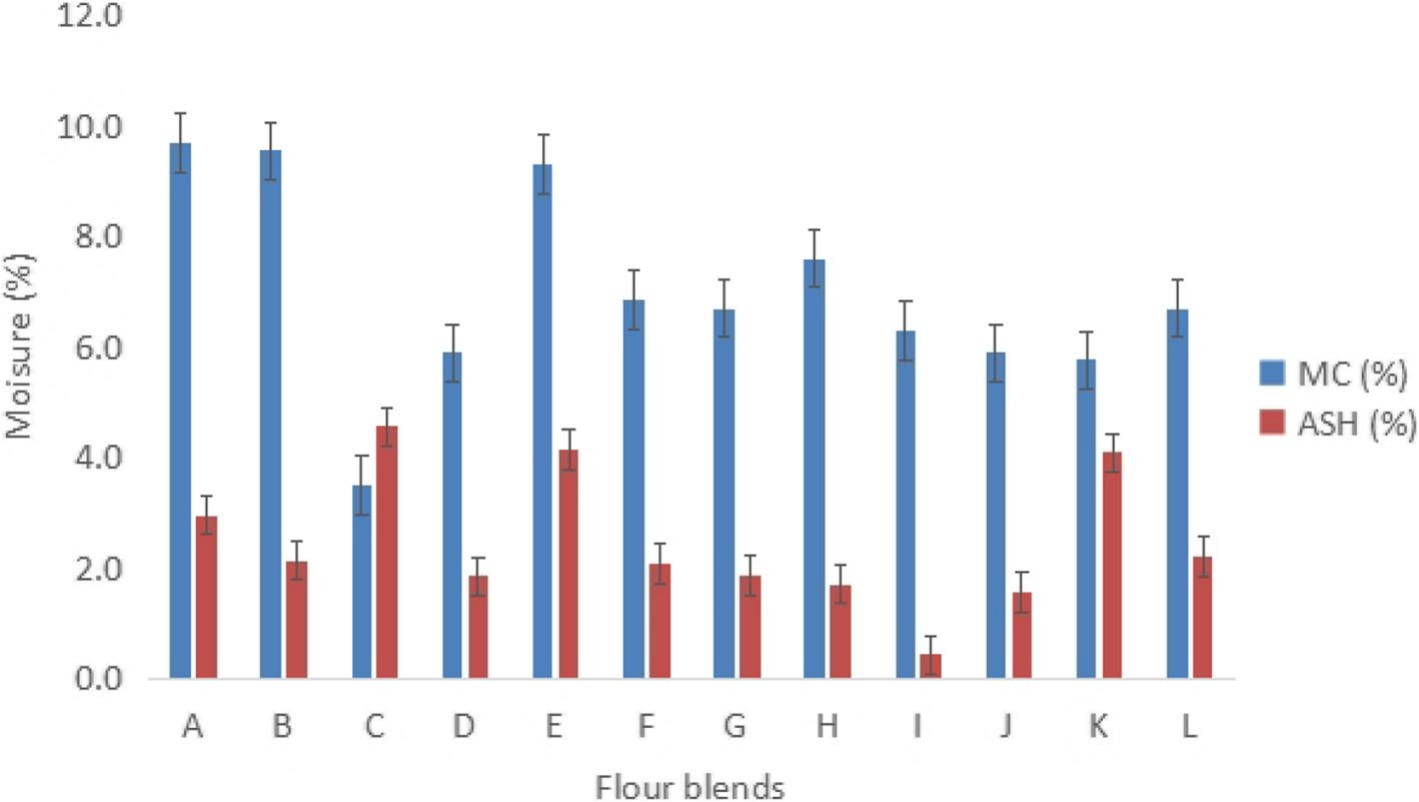
Moisture and Ash content of blended flour from SMEs. MC; Moisture content, A-L; Flour blends from Zambia and Malawian SMEs

**Fig. 2:**
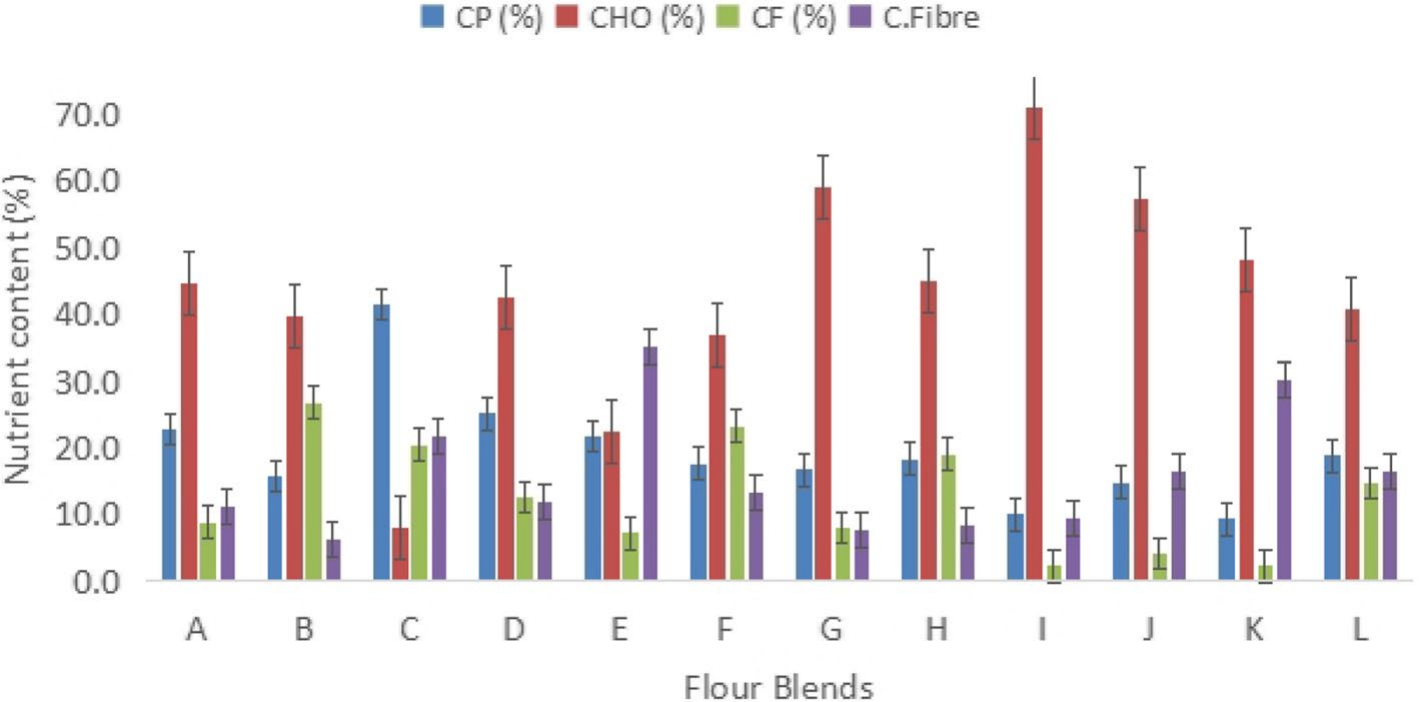
Macronutrients composition of legume blended flours. CP; Crude protein, CHO; Carbohydrates, CF; Crude Fat, C.Fibre; Crude Fibre. A-L; Flour blends from Zambia and Malawian SMEs

**Fig. 3:**
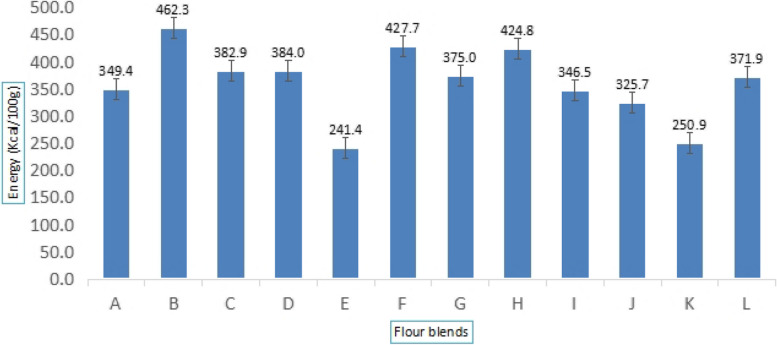
Energy content of flour blend. A-L; Flour blends from Zambia and Malawian SMEs

Proximate composition of the flour blends results is presented in Figs. 1, 2 and 3. The moisture levels in the flour blends showed considerable variation (*p* = 0.05), ranging from 3.5% to 9.70%. However, all blends achieved the minimum moisture content of 10% as recommended by the World Food Programme [28] (see Fig. 1). The ash content also varied significantly (*p* = 0.00), with Blend C recording the highest at 4.56% and Blend L the lowest at 0.44% (see Fig. 1). Crude protein content ranged from 10.13% to 41.55%, with blends containing higher proportions of legumes demonstrating increased protein levels (see Fig. 2).

The fat content varied between 2.33% and 26.77%, with several blends surpassing the 6% fat content found in CSB + (see Fig. 2). Carbohydrate levels were significantly different, ranging from 8.11% in Blend B to 59.0% in Blend A (see Fig. 2). Crude fiber content ranged from 6.20% to 35.20%, with Blend B having the least and Blend E having the most (see Fig. 2).

Energy content varied from 241.4 ± 6.08 to 462.33 ± 5.16 kcal/100 g, and Blends B, F, and J met the recommended minimum energy level of 400 kcal/100 g for supplemental foods (see Fig. 3).

### Micronutrient (Iron and Zinc) composition

**Fig. 4:**
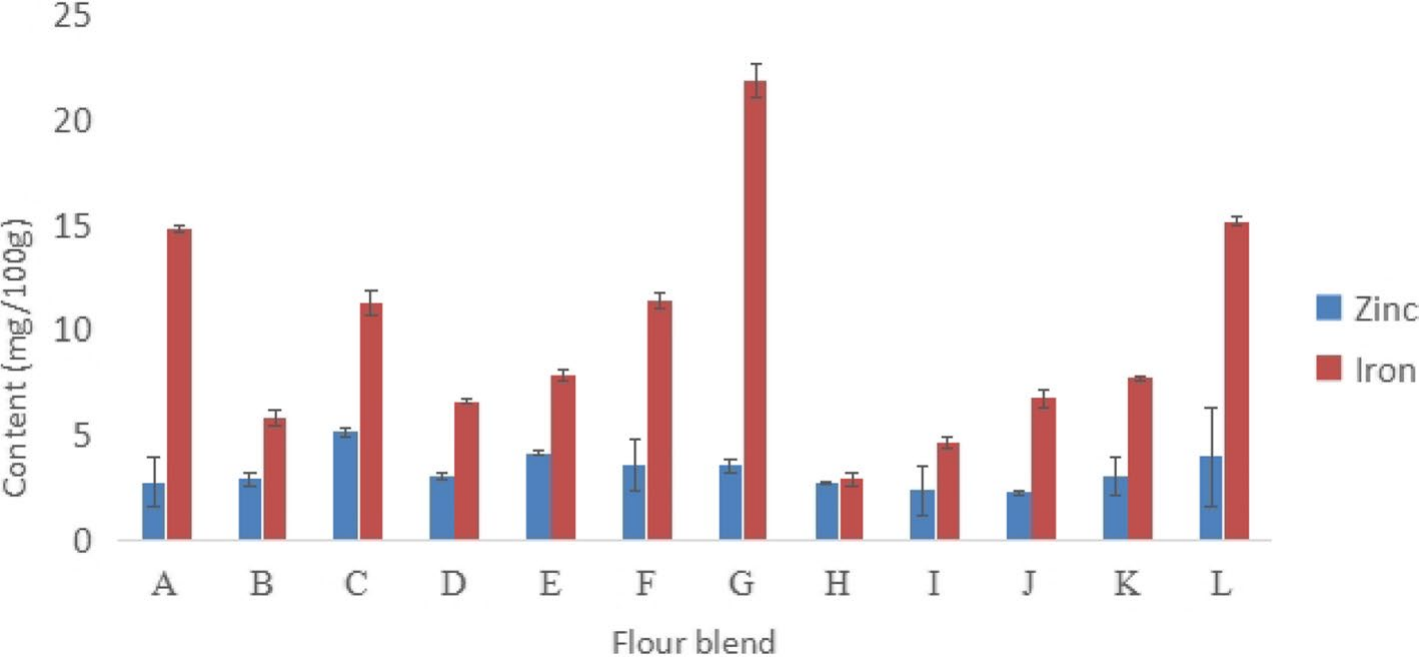
Micronutrient composition of legume-based blended flours. A-L; Flour blends from Zambia and Malawian SMEs

Figure 4 shows micronutrient composition of legume-based flour blends. The study revealed that the iron content ranged from 2.87 ± 0.36 to 21.89 ± 0.83 mg/100 g, while zinc content ranged from 2.24 ± 0.08 to 5.15 ± 0.22 mg/100 g. All blends had zinc levels below the recommended amount for children aged 1–3 years.

### Nutrient provision of SME flour blends vs. CSB + : RDA contribution for children aged 1–3 per two servings

The nutritional composition of blended flours processed by SMEs, the reference corn soy blend plus (CBS +), and the recommended dietary allowance (RDA) for 1–3-year-old children is shown in Table 3. The protein content varied among the flour blends, with blend B containing the highest protein content at 39.8 g, representing 306.4% of the RDA. The lowest protein content was observed in blend J at 9.0 g, providing 69.4% of the RDA. Flour blends H (9.6 g) and J (9.0 g) provided less protein than the recommended 13 g for children aged 1–3 years.

None of the flour blends met the RDA for fat based on two servings. Flour blends K (29.0 g), E (33.8 g) and C (21.0 g) provided more than the RDA for fiber, while all other blends provided less than the required carbohydrates levels. Furthermore, this study revealed that all the blended flours provided less than the recommended daily energy intake of 1331 kcal for children aged 1–3 years. Only flour blends B, F, and H met the energy requirement of 380 kcal/100 g dry weight, while the other blends provided less than this value. The iron content in the flour blends was generally lower than in CBS + , except for three blends. Similarly, zinc content was lower in all the flour blends compared to CBS + . However, flour blends C (5 mg), E(4 mg), F(3.4 mg), G(3.4 mg), and L(3.8 mg) provided more than the RDA for zinc in 1–3-year-old children.

### Functional properties

Results of water Holding Capacity (WHC) and Oil holding capacity (OHC) of blended flours are presented in Table 1. Flour D exhibited highest retention of water of 116.23%. Porridge or foods made from flour blend D would retain more moisture and have a softer and more pleasant texture. The results in Table 1, indicate significant differences in oil holding capacity (OHC) among the flour blends. Flour D had the highest OHC (137.61%), indicating a remarkable ability to retain and absorb oil.

**Table 1:** Relative nutrient provision of flour blends made by SMEs and corn soy blend and their contribution to RDA for 1–3-year-old children

Sample	Ref	Energy		Crude Protein		Crude Fat		CHO		Fiber		Iron		Zinc	
		Intake (Kcal)	% of RDA	Intake (g)	% of RDA	Intake (g)	% of RDA	Intake (g)	% of RDA	Intake (g)	% of RDA	Intake (mg)	% of RDA	Intake (mg)	% of RDA
	RDA^a^	1331.02^e^	100.0	13^d^	100.0	27^f^	100	130^d^	100.0	19^d^	100.0	7^d^	100.0	3^d^	100.0
	CSB + ^b^	364.8	27.4	10.5	80.5	4.1	15.2	61.7	47.5	3.6	18.9	8.6	123.4	8.1^c^	270.0
A		335.6	25.2	15.2	116.6	25.8	95.6	42.8	32.8	10.6	56	14.2	203.6	2.6	88
B		443.8	33.3	39.8	306.4	19.6	72.6	38	29.2	6	31.4	5.6	80	2.8	92.2
C		367.6	27.6	24.2	186	12	44.4	7.8	6	21	110.2	10.8	154.6	5	164.8
D		368.6	27.7	20.8	160.2	7	25.9	40.8	31.4	11.6	60.6	6.4	90.4	2.8	96.6
E		231.8	17.4	16.8	130	22.4	83.0	21.6	16.6	33.8	177.8	7.6	107.6	4	132.8
F		410.6	30.8	16.2	124	7.6	28.1	35.4	27.2	12.8	67.2	11	156.2	3.4	114
G		360	27.0	17.6	135.2	18.4	68.1	56.6	43.6	7.4	39	21	300.2	3.4	114
H		407.8	30.6	9.6	74.6	2.2	8.1	43.2	33.2	8	42	2.8	39.4	2.6	87.4
I		332.6	25.0	14.2	109.2	4	14.8	68.4	52.6	9.2	48.6	4.4	63.2	2.2	74.8
J		312.6	23.5	9	69.4	2.2	8.1	55	42.2	15.6	82.4	6.4	92.4	2.2	71.6
K		240.8	18.1	18.2	139.6	14.2	52.6	46.2	35.6	29	153	7.4	105.6	3	97
L		357	26.8	21.8	168.4	8.6	31.9	39.2	30.2	15.8	83.4	14.6	208.4	3.8	126.8

^a^RDA = Recommended dietary allowance for infants and babies of 1-3years

^b^Corn soy blend flour plus (Standard flour for complementary feeding of infants and children (USDA, 2019) [29] per two servings (96g)

^c^To not exceed the maximum zinc content in 100g

^d^Dietary reference intakes adopted from institute of medicine of the National Academic of Sciences, November 30, 2010

^e^Adopted from the Food and Nutrition Board [30]

^f^RDA for children aged 1–3 years old weighing 12.9kg [31]

*N. D* Not determined

### Microbial characteristics

Results on microbial load of the flour blends are presented in Table 2. There were variations in colony counts among the flour blend indicating differences in microbial contamination levels. Flour blend A exhibited the highest colony count of 560 cfu/g while Flour blend G had the lowest colony count of 22 cfu/g.

**Table 2:** Functional properties of flour

Flour blend	WHC (% + SD)	OHC (% + SD)
**A**	109.78^f^ ± 3.71	80.99abc ± 0.48
**B**	93.79d^e^ ± 2.57	91.31^c^ ± 5.83
**C**	80.11ab^c^ ± 4.02	80.04^ab^ ± 5.87
**D**	116.23^f^ ± 3.65	137.61^e^ ± 1.67
**E**	98.67^e^ ± 0.41	79.16^ab^ ± 3.55
**F**	78.49^ab^ ± 1.68	89.23^bc^ ± 4.60
**G**	109.83^f^ ± 2.87	113.46^d^ ± 1.77
**H**	111.59^f^ ± 4.41	81.76^abc^ ± 0.91
**I**	94.20^de^ ± 0.34	86.62^bc^ ± 3.94
**J**	89.96^de^ ± 2.59	84.86^abc^ ± 1.23
**K**	85.39^bcd^ ± 3.99	74.71^a^ ± 0.43
**L**	94.17^de^ ± 4.32	90.03^bc^ ± 1.82
**M**	73.34^a^ ± 3.48	83.71^abc^ ± 5.17
**N**	88.16^cd^ ± 1.71	81.83^abc^ ± 5.62
	0.00	0.00

Each value is a mean of triplicate % ± SD of triplicate

Means with no common letters within a column significantly differ (*p* < 0.05)

## Discussion

### Proximate composition of flour blends

Flour blends in this study met the minimum moisture content (10%) as recommended by World Food Programme [28]. According to Abay et al. [31], the type of food, the variety of food, and the storage circumstances all affect the moisture content of a food. The flour's low moisture content improves its storage stability by limiting biological reactions and preventing the growth of mold [32]. It can be suggested that the low moisture contents for the flour blends observed in the current investigation would possibly help in increasing the shelf lives of the flour blends or products produced from these flours. However, the moisture content of some flours was unnecessarily too low (Fig. 1), which could affect farmer’s income due to loss of weight or bulkiness contributed by moisture. Water plays a significant role as solvent and filling materials, but also as means to maintain the functions and structure of macromolecules [33–35]. In some cases, a moisture content of less than 14% is advised for safe storage [36].

The results in the study showed that ash content in flour blends C (4.56%) and E (4.10%) was higher than ash content in CSB + , which is 4.1 g/100 g [25], a flour that is consumed by under-five children as part of complementary feeding [28]. A study by Ojo et al. [37] revealed that flours with legumes had higher ash content than those blended with cereal flours due to inherent differences in the composition of legumes and cereal grains. The higher ash content observed in legume-based flour blends can be attributed to their rich mineral profile, which includes calcium, magnesium, phosphorus, potassium, and trace elements such as iron and zinc.

Ash content represents the total mineral content of food, and the current investigation has revealed that flour blend C might be a better source of essential minerals compared to other flour blends. These minerals play a critical role in physiological processes, including maintaining a healthy bone structure, muscle function, nerve signalling, and fluid balance [38]. Additionally, minerals contribute to the formation of hormones, enzymes, and other biologically active compounds in the human body [39].

However, while a higher ash content suggests a richer mineral composition, excessive levels may pose risks, particularly in infants and young children. An imbalance in minerals such as calcium and phosphorus could lead to potential toxicity or interference with nutrient absorption. Moreover, flours with an ash content exceeding recommended guidelines might not comply with nutritional standards for corn-soy blend flours as outlined by the World Food Programme [28]. Therefore, while the incorporation of legumes in flour blends enhances mineral content, it is essential to ensure that the overall composition aligns with established nutritional guidelines to support optimal growth in under-five children.

Furthermore, ash content can also serve as a quality indicator for contamination in food samples [40]. Higher ash levels may indicate the presence of undesirable inorganic substances, such as soil, sand, or contaminants introduced during processing. Therefore, while evaluating ash content as a nutritional parameter, it is also crucial to assess food safety and processing standards to ensure the quality and safety of complementary foods.

The results in this study align with findings by Godswil [41], where the crude protein content of flour blends ranged from 16.32% to 44.10%. Most flour blends examined in this study contained higher protein levels than the 14 g/100 g found in CSB + , as recommended by the World Food Programme [28]. The elevated protein content in certain flour blends may be attributed to a greater proportion of legumes such as soybeans, which significantly contribute to the overall protein concentration. While increasing protein content in complementary foods is encouraged for improving child nutrition, it is essential to consider the economic and health implications. Flour blends with higher legume content, such as blend C, may be more expensive due to the cost of incorporating protein-rich ingredients. This could lead to higher production costs, increased prices, and reduced affordability for low-income households. Furthermore, excessive protein intake during infancy has been associated with accelerated growth and an increased risk of overweight and obesity later in life, despite protein’s essential role in muscle development and overall growth [42–45].

Therefore, while formulating complementary foods, it is crucial to strike a balance between enhancing protein content and ensuring affordability and compliance with nutritional guidelines. Moderate inclusion of legumes can help maintain an optimal protein level without excessive cost burdens. Additionally, adherence to dietary recommendations can help mitigate the risk of long-term metabolic disorders associated with excessive protein intake. As such, careful formulation and cost-effective strategies should be considered to provide accessible and nutritionally balanced complementary foods for under-five children.

The fat content in blended flours can influence the texture and mouthfeel of porridge, which directly affects its acceptability among infants. While some children may enjoy a richer, higher-fat porridge, others may find it too heavy. The variation in fat content among flour blends can largely be attributed to the type and quantity of legumes incorporated. Legumes such as soybeans and groundnuts have a naturally high fat content, which significantly increases the overall fat percentage in flour blends compared to legumes like beans and peas. Myzia et al. [46] demonstrated that fat content in malted sorghum flour increased from 2.59% to 9.26% with the inclusion of soy flour, highlighting the impact of legume selection.

Including legumes in cereal-based flours can enhance their fat content, providing essential fatty acids necessary for growth and development. Fats play a crucial role in maintaining bodily structures, regulating metabolism, and serving as a secondary energy source [47]. However, excessive fat intake in children can lead to obesity, which is associated with health risks such as high blood pressure, high cholesterol, impaired glucose tolerance, type 2 diabetes, asthma, and musculoskeletal complications [48]. Additionally, obesity has been linked to psychosocial issues, including depression and social difficulties [49, 50].

Carbohydrate content in flour blends is influenced by the proportion of cereals included, with maize-based blends typically having higher carbohydrate levels. Flour blend A, for instance, may be more suitable for children requiring a higher carbohydrate intake to support energy needs. Conversely, flour blends with lower carbohydrate content may be more appropriate for children who require reduced carbohydrate intake for specific dietary needs. Carbohydrates are essential for providing energy and contribute to the taste, texture, and overall appeal of food products [51, 52]. For children's consumption, it is recommended to select flour blends based on their specific nutritional needs. Flour blends incorporating soy or groundnut flour can be beneficial for children requiring higher fat and protein intake for growth and development. However, moderation is necessary to prevent excessive fat intake. Cereal-based flour blends with balanced carbohydrate content, such as those containing maize and sorghum, are ideal for children with high energy needs. Blends with legumes like beans and peas provide a more moderate fat content while still supplying essential nutrients, making them a suitable option for general complementary feeding. By carefully selecting flour blends, caregivers can ensure optimal nutrition for children's growth and development.

The observed differences in energy content can be attributed to the proportion of legumes and cereals incorporated into each blend. Legume-rich formulations, particularly those containing soybeans and groundnuts, contribute to higher energy values due to their elevated fat and protein content [53]. Conversely, blends with a higher proportion of cereals, such as maize and sorghum, tend to have lower energy content, primarily due to their carbohydrate composition, which provides energy at a relatively lower caloric density compared to fats [54].

Ensuring that complementary flour blends meet the recommended energy levels is crucial for addressing the nutritional needs of young children, particularly those at risk of malnutrition. Energy-dense blends such as B, F, and J provide sufficient caloric intake necessary for growth and development. However, for formulations with lower energy content, fortification strategies or modifications in ingredient composition may be necessary to enhance their caloric value [55].

Additionally, the balance of macronutrients within these flour blends is important for achieving optimal energy utilization. While fat-rich legume blends offer higher energy density, excessive fat content may affect acceptability and digestibility, particularly in infants [56]. Therefore, an appropriate balance between legume and cereal components should be maintained to ensure that the blends are both nutritionally adequate and suitable for regular consumption [57].

Dietary fiber is well known for its links to a favourable body weight, a healthy gut flora, a good overall metabolic state, and a lower risk of cardiovascular disease and mortality [58]. Consuming a diet rich in dietary fiber has numerous health implications, both positive and potentially negative, depending on various factors such as individual health status, fiber type, and overall dietary patterns [59, 60]. Although fiber helps in constipation prevention, lowering risk of chronic diseases and colorectal cancer, regulating blood sugar levels, cholesterol reduction, contribute to weight management, and, it has some negative implications such as digestive discomfort, and reduced absorption of minerals such as calcium, iron, zinc and magnesium [61, 62].

According to Codex Alimentarius, [62], the three blended flour B (462.33 kcal/100 g), F (427.7 kcal/100 g), and J (424.84 kcal/100 g)—met the 400 kcal/100 g or 4 kcal per g of flour minimum recommended energy for supplemental food. The energy content of the flours was enough for feeding infants between the ages of six and twenty-three months on a complementary diet consisting of two meals daily plus breast milk. Other flour blends with less than 400 kcal/100 g did not meet the minimum energy requirement as supplemental food (Fig. 4). This means that children who could depend on porridges made from these flour blends would not meet the recommended minimum energy requirement and may be undernourished.

### Micronutrient (Iron and Zinc) composition

All flour blends had zinc contents that was below the recommended amount for 1–3-year-old children [28, 63]. Iron and zinc are essential micronutrients, and their presence in legume-based flours can contribute to enhancing the nutritional value of various food products [64]. The body uses iron primarily for two essential processes: generating energy and transporting oxygen. On the other hand, zinc also has physiological effects on the immune system and enzymatic processes [65]. The difference in iron and zinc concentration among different flour blended samples may, therefore, have an impact on the nutritional value of foods made from these flours. Due to wide range of iron concentration, selection of proper flours can have a significant impact on iron status of children. In this regard, flour blend H, having highest iron concentration, could be an ideal choice for children and pregnant women who are at risk of iron-deficiency anemia [66]. Flour C has the highest zinc concentration, possibly making it a better choice for children trying to increase their dietary intake of zinc.

### Nutrient provision of SME flour blends vs. CSB + : RDA contribution for children aged 1–3 per two servings

#### Proximate composition

It is essential that children consume adequate proteins for their proper growth and development of their bodies to produce new tissues and repair damaged ones. Proteins are essential in the synthesis of enzymes, hormones, and other vital bodily functions [67]. Similarly, flour blends H and J manufactured by SMEs would provide less protein than the standard CSB that the WHO recommends for infant feeding. This implies that these blends would be less preferred on the market making it less likely for the SMEs to penetrate the market and build a successful business. Likewise, high protein content of sample could come from disproportionately high level of legume incorporation in the blend making the flour unnecessarily expensive since legumes are more expensive than cereals. High protein content in the flour blends could come from legumes such as soybeans [68]. To recover for cost of legumes ingredients, most flour blends would be sold at a higher price making it less saleable on the market and eventually force SMEs out of business. Undernutrition in infants may result from inadequate protein intake during supplemental feeding [69], whereas overconsumption of protein may increase the risk of obesity and being overweight later in life [70].

Infants and early children should consume fat in their diets because it has a high energy density and helps the body absorb fat-soluble vitamins [71]. However, too much fat intake may lead to obesity in children which can result into cardiovascular diseases such as hypertension, type 2 diabetes and breathing problems [72]. Fat also offers vital polyunsaturated fatty acids such as omega-3 and omega-6, which are necessary for healthy brain development in infants and children [73].

All blended flours would provide less energy than recommended energy of 1331 kcal/day for a 1–3-year-old child when two servings could be provided as they only contribute less than 40% of the RDA. The data shows that if children could be given two servings of these flour blends, which is a common practice in resource poor households, children would be getting less than their RDA. Overall, apart from flour blends B, F and H, the rest of the flour blends would provide less than 380 kcal/100 g dry weight of the standard corn soy flour [29]. These results are similar to what Makame et al., [74] found for 8 common locally African complementary porridges that did not meet the energy needs of children. The body of a child needs energy to sustain vital processes such as blood circulation, respiration, and cell development and repair [30]. Though the fiber contents of flour blends C (21.0 g), E (33.8 g), and K (29.0 g) were generally higher than those of CSB + and RDA, the rest of the flour blends fell short to meet the RDA for children between the ages of 1–3 years after two servings (Table 3). The fiber contents ranged from 6.0 to 33.8 g, meeting 31.4% to 177.8% of the RDA for children aged 1 to 3 years.

**Table 3:** Microbial analysis of legume-based flour blends

Flour blend	TVCC (10^2^ cfu/g)
A	5.6
B	3.45
C	1.39
D	0.66
E	4.14
F	1.93
G	0.22
H	2.45
I	TNTC
J	TNTC
K	8.83
L	1.58

*TNTC* Too numerous to count

*TVC *Total viable colony count

Each value is a mean of duplicate

Iron and zinc are the two most critical micronutrients for public health in developing countries [50]. Iron is a mineral that is generally present in a wide variety of foods, but it can also be provided to infants and children as dietary supplements. Iron is an essential component of hemoglobin, an erythrocyte (red blood cell) protein that delivers oxygen from the lungs to the tissues [75]. A lack of iron in the first few years of life can cause a variety of cognitive, emotional, and social issues in children [76]. Iron deficiency affects 20% to 50% of the global population, and it causes half of all episodes of anemia in children. A study conducted in Nigeria by Ezeokeke & Onuoha, [77] aimed to determine the nutritional makeup of flour blends made from maize (cereal), soybean (legume), and banana (fruit) as supplemental diets for older babies found contradicting results. They found that zinc and iron levels ranged from 2.7 to 4.55 mg/100 g, and 3.34 to 15.36 mg/100 g, respectively. The study discovered that blended flours had significantly less zinc and iron (*p* < 0.05) than the control sample (Celerac). Another study conducted in Kenya by Liomba et al., [78] aimed at improving the iron and zinc contents of the usual traditional maize-based complementary porridge by blending with high energy and micronutrient foods revealed that all flour blends met the iron and zinc recommendations for infants. Nevertheless, too much iron intake by children has been associated with poisoning leading to death of children [79]. Similarly, too much intake of zinc above the Upper tolerance levels may lead to zinc toxicity in children [80].

#### Functional properties

WHC and OHC are critical components that influence the consistency, flavor, and overall quality of food products, especially those that are consumed by children [81]. Porridge or foods made from flour blend D would retain more moisture and have a softer and more pleasant texture. Therefore, it is expected that consumption and acceptability of such porridges would be increased and consequently leading to infants and young children receiving essential nutrients the porridge contains.

Flours blends K demonstrated low WHC (73.34%), indicating that it has a weaker capacity to retain water which may affect its physical and sensory properties. Porridge or any product made from this flour might be drier, crumblier, or less moist, which might affect the appeal to children. Young children are less likely to enjoy dry and unpleasant food textures, which could result in a reduction in the ingestion of nutrient-dense foods [82]. The variance in WHC among these flours emphasizes how crucial ingredient choice is when creating items for young children. When developing recipes for infant cereals, snacks, and other children-targeted goods, food processors, nutritionists, dieticians must consider ultimate WHC of flours blends and strive to strike a balance between texture and nutrient content of the final product.

Porridges made with flour D may retain more moisture and contain more oil, giving them a richer and more pleasant texture. This may be advantageous to children since it may enhance the flavour and general acceptance of the foods, thereby increasing consumption. Therefore, flour blend D might be considered as a good option for developing tasty, nutrient-dense, and appealing foods for infants and young children.

In contrast, flour K displayed a substantially lower OHC value (74.71%) than the other flours examined, demonstrating a reduced capacity to absorb and hold oil. The sensory properties of porridges or food products made with flour K may be negatively affected leading to less preference or appeal by children. Consequently, young children may consume fewer essential nutrients because they are less likely to enjoy the flavour and texture of flour blend. The differences in OHC can be attributed to several factors, including the composition and structure of the legumes from which the blend was made from [83]. Legumes contain different components, including proteins, fibers, starches, and lipids, which can interact differently with oil and influence the oil holding capacity [84]. The observed variations in OHC among the flours have implications for their potential applications in food products. Flours with lower OHC values, like flour K, may be preferred for products where limited oil absorption is required, such as low-fat formulations or oil free preparations [85].

#### Microbial characteristics

Flours with microbial colony counts exceeding 100 cfu/g are considered unsafe for infant and child feeding, as high colony counts can indicate potential food safety concerns [86]. The presence of excessively high colony counts in some flours suggests that these products could pose significant risks to children’s health, including foodborne illnesses. In extreme cases, the microbial load was so high that counting the individual colonies became nearly impossible, pointing to unsafe food handling practices and poor hygienic conditions during production, storage, or transportation. These high microbial counts, often indicating contamination with bacteria and fungi, could negatively impact both the safety and nutritional quality of the flours.

The total viable colony count (TVC) serves as an important indicator of microbial contamination in food. A high TVC suggests poor hygiene during the production and storage phases, while lower TVC values indicate effective microbial control and sanitation [87]. For complementary flours, which are typically consumed by vulnerable populations such as infants and young children, any microbial contamination poses a heightened risk to health, as children’s immune systems are not yet fully developed to combat infections from harmful microorganisms [88]. Thus, maintaining strict hygiene and quality control during all stages of flour production, storage, and handling is essential to ensure food safety and to prevent health risks.

Excessive microbial contamination in flour can lead to the spoilage and deterioration of its nutritional quality, which is particularly concerning for complementary foods that are intended to support the growth and development of young children [89]. The nutritional loss, particularly in terms of essential vitamins and proteins, could exacerbate malnutrition or lead to deficiencies in children, who are particularly vulnerable to the effects of poor nutrition. Microbial contamination in legume-based flours can occur at multiple stages of the food production process, including during the production, harvest, processing, and storage phases [90]. Environmental factors such as humidity, temperature, and air quality during these stages can influence the proliferation of microorganisms. Furthermore, inadequate handling and storage practices, such as poor packaging or insufficient refrigeration, can exacerbate microbial growth. Therefore, improving hygienic practices across small and medium-sized enterprises (SMEs) producing flour blends is critical for reducing microbial contamination and ensuring the safety of complementary foods intended for children.

## Conclusion

The study highlights that flour blends produced by SMEs in Malawi and Zambia have potential as sources of essential nutrients but often lack proper technical guidance in formulation. Variations in nutrient composition were observed, with some blends containing excessive legumes, resulting in high protein and fiber levels, which disrupt the desired nutrient balance. While these blends can provide important nutrients, they fall short of meeting the Recommended Dietary Allowances (RDA) for children aged 1–3 years when consumed at the typical daily intake of 96–100 g. Since no single food can meet all nutritional needs, it is crucial for SMEs to align their products with the nutrient profiles of CSB + to remain competitive in the market. Additionally, some blends exhibited concerning microbial contamination, indicating inadequate hygienic practices. To improve product quality and safety, SMEs should standardize nutrient content and enhance hygiene practices across production, packaging, and distribution processes. This study emphasizes the need for careful formulation of legume-based flour blends to ensure they meet the nutritional requirements of young children, especially in regions affected by undernutrition.

### Recommendations

1. It is recommended that SMEs should ensure that they consider optimization formulations to achieve the desired nutrient levels without significantly increasing production costs.
2. SMEs must explore cost-effective ways in managing protein content in blended flours balancing nutritional benefits and production costs.
3. SMEs should ensure hygiene and manufacturing practices that can help in microbial load reduction in blended flours are adhered during processing.

## Nomenclature

PEM Protein energy malnutrition

PLWHA People living with HIV and AIDS

SMEs Small and Medium Enterprises

LUANAR Lilongwe Agriculture and Natural Resources

AOAC Association of Official Agricultural Chemists

CHO Carbohydrates

WHC Water Holding Capacity

OHC Oil Holding Capacity

ANOVA Analysis of Variance

LSD Least Significant Difference

TVC Total viable Count

CSB+ Corn soy blend plus

RDA Recommended dietary Allowance

UNZA University of Zambia

WFP World Food Programme

NA Nutrient Agar

USAID United States Agency for International development

AAS Atomic absorption spectrometry 

PUFA Poly unsaturated Fatty Acids

WHO World Health Organization

cfu Colony forming units

Kcal Kilocalories

TNTC Too numerous to count

CVD Cardiovascular diseases

SDGs Sustainable Development Goals

## Data Availability

No datasets were generated or analysed during the current study.
